# The Strawberry FaWRKY1 Transcription Factor Negatively Regulates Resistance to *Colletotrichum acutatum* in Fruit Upon Infection

**DOI:** 10.3389/fpls.2019.00480

**Published:** 2019-04-18

**Authors:** José Javier Higuera, José Garrido-Gala, Ayman Lekhbou, Isabel Arjona-Girona, Francisco Amil-Ruiz, José A. Mercado, Fernando Pliego-Alfaro, Juan Muñoz-Blanco, Carlos J. López-Herrera, José L. Caballero

**Affiliations:** ^1^Departamento de Bioquímica y Biología Molecular, Campus de Excelencia Internacional Agroalimentario ceiA3, Universidad de Córdoba, Córdoba, Spain; ^2^Departamento de Protección de Cultivos, Instituto de Agricultura Sostenible (CSIC), Córdoba, Spain; ^3^Unidad de Bioinformática, Servicio Central de Apoyo a la Investigación (SCAI), Universidad de Córdoba, Córdoba, Spain; ^4^Departamento de Biologia Vegetal, Universidad de Málaga, Málaga, Spain

**Keywords:** WRKY transcription factor, *Colletotrichum acutatum*, *Agrobacterium* transformation, strawberry fruit defense response, *Fragaria* × *ananassa*

## Abstract

Strawberry (*Fragaria* ×*ananassa*) is a major food crop worldwide, due to the flavor, aroma and health benefits of the fruit, but its productivity and quality are seriously limited by a large variety of phytopathogens, including *Colletotrichum* spp. So far, key factors regulating strawberry immune response remain unknown. The *FaWRKY1* gene has been previously proposed as an important element mediating defense responses in strawberry to *Colletotrichum acutatum*. To get further insight into the functional role that FaWRKY1 plays in the defense mechanism, *Agrobacterium*-mediated transient transformation was used both to silence and overexpress the *FaWRKY1* gene in strawberry fruits (*Fragaria* ×*ananassa* cv. Primoris), which were later analyzed upon *C. acutatum* inoculation. Susceptibility tests were performed after pathogen infection comparing the severity of disease between the two agroinfiltrated opposite halves of the same fruit, one half bearing a construct either for *FaWRKY1* overexpression or RNAi-mediated silencing and the other half bearing the empty vector, as control. The severity of tissue damage was monitored and found to be visibly reduced at five days after pathogen inoculation in the fruit half where *FaWRKY1* was transiently silenced compared to that of the opposite control half and statistical analysis corroborated a significant reduction in disease susceptibility. Contrarily, a similar level of susceptibility was found when *FaWRKY1* overexpression and control fruit samples, was compared. These results unravel a negative regulatory role of FaWRKY1 in resistance to the phytopathogenic fungus *C. acutatum* in strawberry fruit and contrast with the previous role described for this gene in *Arabidopsis* as positive regulator of resistance against the bacteria *Pseudomonas syringae*. Based on previous results, a tentative working model for WRKY75 like genes after pathogen infection is proposed and the expression pattern of potential downstream FaWRKY1 target genes was also analyzed in strawberry fruit upon *C. acutatum* infection. Our results highlight that FaWRKY1 might display different function according to species, plant tissue and/or type of pathogen and underline the intricate FaWRKY1 responsive defense regulatory mechanism taking place in strawberry against this important crop pathogen.

## Introduction

Strawberry has grown in importance throughout the world, due to both the fact that this small fruit has become a highly relevant product at the social level for its nutritional properties and beneficial effects on health (Giampieri et al., 2017; Garrido-Bigotes et al., 2018a), and its economic importance, being one of the products with the largest share in the export of fruits and vegetables^1^.

Strawberry exhibits wide diversity in its susceptibility to a large variety of phytopathogenic organisms, including *Colletotrichum* spp. causal agent of anthracnose, a major disease of this crop (Simpson, 1991; Jeger and Bailey, 1992; Maas, 1998), yielding major losses in fruit production at the pre-harvest stage (Guidarelli et al., 2011). Three *Colletotrichum* species cause the anthracnose diseases of strawberry: *C. fragariae* and *C. gloeosporioides* induce the crown rot and lesions in vegetative tissues, while *C. acutatum* is the main pathogen causing the strawberry fruit rot (Peres et al., 2005). Anthracnose on strawberry is found worldwide and is a very destructive disease, causing up to 80% of plant death in nurseries and over 50% of yield losses in fields (Sreenivasaprasad and Talhinhas, 2005). Fungicide applications are resource-demanding every year for this and other important crops, to secure production yield but this increases public concern for environment food safety and makes urgent the need to develop sustainable alternatives.

*Colletotrichum* spp. is considered an hemibiotrophic pathogen and the histopathology of the interaction strawberry-*C. acutatum* has been previously well reported and monitored by using light, fluorescent and transmission electron microscopy with a symptomless, brief biotrophic phase, preceding a main necrotrophic development within the strawberry tissues and the rise of lesions (Curry et al., 2002; Horowitz et al., 2002; Peres et al., 2005; Amil-Ruiz et al., 2016). Although much research has been dedicated to understand the interplay between fungal pathogen and plant, there is a lack of comprehensive information on the molecular level and mechanisms underlying the process of defense and resistance to this pathogen in strawberry. Thus, characterizing the strawberry defense-responsive components will lead to improve the understanding of the underlying molecular mechanisms of defense. Indeed, it represents a major piece within the strategies to improve resistance in this important crop, which is a relevant economic and environmental issue.

The resistance to invaders is frequently harmonized in plant through a complex defense molecular network fine-tuned by phytohormones such as salicylic acid (SA), jasmonic acid (JA), and ethylene (ET), mainly, but also abscisic acid (ABA) and gibberellins (GA), which regulate the defensive response to efficiently face the different pathogens (Robert-Seilaniantz et al., 2011). It is well known that SA signaling pathway is mainly activated against biotrophic pathogens, and often induces a hypersensitive response (HR) followed by the onset of Systemic Acquired Resistance (SAR) (Fu and Dong, 2013). On the contrary, JA/ET signaling pathway is commonly activated in plant against necrotrophic pathogens, insect, or in response to wounding (Yan and Xie, 2015). JA induces a different set of defense response genes and the production of a large variety of secondary metabolites such as alkaloids, phenolic compounds and terpenes (Zhou and Memelink, 2016). Crosstalk among these signaling pathways has been well described in models (Robert-Seilaniantz et al., 2011) while remains largely unknown or poorly understood in many crop species.

Transcription factors (TFs) are key regulators of gene expression, which play important roles within this complex defense molecular network leading to plant immunity. To date, many defense-related TFs have been identified in plants, including MYBs, the TGA/bZIP family protein, AP2/ERF-ET responsive element binding factors, NACs, the Whirly (WHY) family protein, and WRKYs (Desveaux et al., 2005; Dubos et al., 2010; Seo et al., 2015).

The WRKY family is well known to mediate defense in plant in response to biotic and abiotic stresses but also are involved in other processes such as senescence, seed dormancy and development (Robatzek and Somssich, 2002; Rushton et al., 2010; Liu et al., 2016). The WRKY TF family has been well characterized in *A. thaliana*, comprising 74 members. Most of them are responsive to pathogen infection or signal molecules (Pandey and Somssich, 2009), modulating either positively or negatively the plant defense responses (Eulgem and Somssich, 2007). Many WRKYs have been described as positive regulators of SA-dependent responses in *Arabidopsis* (*AtWRKY18, AtWRKY38, AtWRKY53, AtWRKY54, AtWRKY58, AtWRKY59, AtWRKY66, and AtWRKY70*), being up-regulated during the NPR1-dependent SAR activation (Wang et al., 2006; Ishihama and Yoshioka, 2012). However, WRKY TFs often exhibit a dual activity in plant defense, depending on the type of pathogen. For instance, AtWRKY70 plays an important role as integrating signals from SA- and JA- dependent response, being responsible for inducing SA-responsive PR genes to enhance the resistance to biotrophic pathogens, at the time that repress the expression of JA-responsive genes, compromising resistance to necrotrophs in vegetative tissue (Li et al., 2004; Li et al., 2006). Similarly, AtWRKY50 and AtWRKY51 act as positive regulators of SA-mediated signaling, as well as negative regulators of JA-mediated signaling in *Arabidopsis* leaves (Gao et al., 2011). Also in *Arabidopsis* vegetative tissues, AtWRKY3, and AtWRKY4, two structurally similar WRKYs, have been described as positive regulators of plant resistance to necrotrophic pathogens such as *Botrytis cinerea*, but AtWRKY4 negatively affects the resistance to biotrophic pathogens such as *Pseudomonas syringae* (Lai et al., 2008). Also, constitutive expression of *AtWRKY33* conferred increased resistance to fungal necrotrophic pathogens, but enhanced susceptibility to the bacterial pathogen *P. syringae* (Zheng et al., 2006). On the contrary, the overexpression of *VvWRKY52* in *A. thaliana* green tissues enhanced resistance to biotrophic fungi *Erysiphe cichoracearum* and *P. syringae* pv. *tomato* DC3000 (*Pst* DC3000), but increased susceptibility to the necrotrophic pathogen *B. cinerea* (Wang et al., 2017).

Although many members of the WRKY gene family have been extensively studied in model plants using vegetative tissues, little is known about their defense-related function and regulation in strawberry, particularly in fruit. Strawberry fruit ripening changes the hormonal balance over time of auxins, ABA and GA, among others (Symons et al., 2012), with potential crosstalk effects on the main SA- and JA/ET- defense pathways (Pieterse et al., 2012) and differential expression of genes involved in both constitutive and induced defense mechanisms (Guidarelli et al., 2011). To date, 33 out of 62 *FvWRKY* TFs has been reported to be differentially regulated in the wild diploid strawberry species *Fragaria. vesca* in response to powdery mildew infection (Wei et al., 2016).

*FaWRKY1* was the first strawberry WRKY TF identified as mediator of defense response against to *C. acutatum* in cultivated strawberry (Encinas-Villarejo et al., 2009). *FaWRKY1* encodes an AtWRKY75-like transcription factor type IIc, which is up-regulated after *Colletotrichum* infection and responds to defense-related hormones such as SA, JA, ABA and wounding, being its expression dependent on strawberry cultivar and tissue (Encinas-Villarejo et al., 2009; Amil-Ruiz et al., 2016). In an attempt to characterize the function of this gene within the strawberry defense mechanism we previously undertook the heterologous overexpression of the *FaWRKY1* in *A. thaliana Atwrky75* mutant and wild type (Encinas-Villarejo et al., 2009). The overexpression of *FaWRKY1* in the *Atwrky75* insertional mutant reverted the enhanced susceptible phenotype of the mutant, and even increased resistance over the wild type to avirulent strains of *Pst* DC3000. This resistant phenotype was associated with a strong oxidative burst and glutathione-S-transferase (GST) induction and was uncoupled to pathogenesis-related (PR) gene expression. These results proved for the first time a role of *FaWRKY1* gene in defense response and demonstrated that this strawberry gene could act as a positive regulator of resistance during compatible and incompatible interactions of a gram-negative phytopathogenic bacteria, in a heterologous plant system, pointing out a relevant role of this gene in the defense mechanism of strawberry. However, in strawberry, high level of *FaWRKY1* expression positively correlated with high degree of fruit infection by *C. acutatum* (Encinas-Villarejo et al., 2009). The observed differences between *Arabidopsis* and strawberry could reflect a distinctive modulation of FaWRKY1 biological function in different plant species and/or plant tissues or against different phytopathogens. Recently, the WRKY75 ortholog in apple and a rose were found upregulated in leaves in response to *Alternaria alternata* (Zhu et al., 2017) and *Podosphaera pannosa* and *Diplocarpon rosae* (Neu et al., 2019), respectively, pointing out a role of the WRKY75-like TFs in defense responses on Rosaceae species, irrespectively of the pathogen’s lifestyle. It is worthwhile to note that *AtWRKY75*-like genes also have been described to act differently according to the pathogen lifestyle. Thus, overexpression of *VvWRKY1* increased the resistance of grapevine to the biotrophic pathogen *Plasmopara viticola* (Marchive et al., 2013). However, the *GbWRKY1* acted as a negative regulator of the JA-mediated defense response in cotton and the silencing of this gene resulted in increased resistance to the necrotrophic *B. cinerea* and the hemibiotrophic *Verticillium dahliae* (Li et al., 2014).

To get further insight into the biological role that *FaWRKY1* gene plays within the mechanism of resistance to pathogens in strawberry, we have transiently silenced and overexpressed this gene in strawberry fruit using a modified *Agrobacterium*-mediated transient transformation methodology. Results demonstrate that susceptibility to *C. acutatum*, is significantly reduced in strawberry fruit where *FaWRKY1* was transiently silenced whereas its ectopic overexpression does not substantially change susceptibility to this pathogen. Our study unravels a biologically relevant function of FaWRKY1 as negative regulator of resistance to *C. acutatum* infection in strawberry fruit and contrast with the previous role as a positive regulator of resistance found after its ectopic expression in *Arabidopsis*. Also, the expression pattern of some potential *FaWRKY1* target genes was analyzed. Taken together, results shed light into the intricate FaWRKY1 regulatory network of strawberry fruit defense response against *C. acutatum*.

## Materials and Methods

### Fungal and Plant Material

*Colletotrichum acutatum* isolate CECT 20240 was maintained on potato dextrose agar (Duchefa) at 20°C with 16/8 photoperiod, or grown in strawberry agar (500 g/L grinded strawberry red fruits, 1.5% bacteriological agar) to increase the infectivity prior to pathogen inoculation. Stock conidia suspensions (10^6^ conidia/mL) were prepared by scraping the surface of four-week old mycelia, in sterile distilled water containing 0.03% (v/v) Tween-80, then filtered in glass wool previously to the quantification the conidia concentration with a Neubauer Chamber Cell Counting. For pathogen inoculations, diluted 10^5^ conidia/mL suspensions were prepared.

Strawberry fruits (*Fragaria × ananass*a cv. Primoris) were grown under field conditions in Huelva (Finca Experimental “El Cebollar”, IFAPA), in southwestern Spain.

### Plasmid Construction for Strawberry Fruit Transformation

All amplified sequences and specific primers used for plasmid constructs are described in Supporting Information Supplementary Table S1. Binary plasmids (pK7WG2.0 and pKGWFS7.0) were obtained from VIB Plant Systems Biology (Belgium). pFRN binary vector was courtesy of Dr. Marten Denekamp, Department of Molecular Cell Biology, University of Utrecht (Netherlands). For all the cloning steps using gateway technology, standard Invitrogen protocols were used.

For the transient overexpression of the *FaWRKY1* gene in strawberry fruits, the plasmid pK7WG2::FaWRKY1 (35S::FaWRKY1) previously described in Encinas-Villarejo et al. (2009) was used. For the spatial localization and time course visualization of the transgene after agroinfiltration in strawberry fruits following this innovative procedure, a 1035 bp DNA fragment carrying the complete CaMV35s promoter was specifically amplified from pK7WG2.0 vector with primers p35S-attBfw and p35S-attBrv and cloned into the pDNOR221 entry vector. This 1035 bp DNA fragment was later transferred to pKGWFS7.0 destination vector to obtain the pKGWFS7::pCaMV35s::GUS (p35S::GUS) plasmid derivative where the β-glucuronidase gene is driven under control of the CaMV35 promoter.

The silencing FaWRKY1-RNAi cassette was constructed as follows: a 272 bp non-conserved region of this gene corresponding to the 91 first amino-acids was PCR amplified from pK7WG2::FaWRKY1 with specific WRKY1-RNAi forward and reverse primers and cloned into pCR8/GW/TOPO (Invitrogen) as an entry vector. The cloned fragment was subsequently transferred to the destination pFRN vector to obtain the RNAi silencing construct, pFRN::FaWRKY1-RNAi. The correct sense and antisense orientation of the 272 bp WRKY1 DNA fragment spaced by the CHS intron was confirmed by sequencing prior further manipulations.

All these constructs, including their corresponding empty vectors, were introduced into *Agrobacterium tumefaciens* strain AGL0 (Lazo et al., 1991) using the freeze–thaw shock method (Holsters et al., 1978). *A. tumefaciens* strains were grown at 28°C in Luria–Bertani (LB) medium with appropriate antibiotics. When the culture reached an optical density of about 0.8 at 600 nm (OD600), cells were harvested and resuspended in a modified MacConkey agar (MMA) medium (Spolaore et al., 2001). After 1 h of incubation at 22°C in dark, *Agrobacterium* suspensions were injected into fruits using one-milliliter syringes.

### Fruit Agroinfiltration and Experimental Design

Strawberry fruits at pink/turning stage were collected with a pigmentation degree of approximately 25% (Aharoni et al., 2002). All fruits were excised along with their pedicels, of ten centimeters long, then sterilized with commercial bleach (1:60 v/v) and cultured by pedicel immersion in sterile rich medium MS (0.25 × Murashige Skoog and 4 g sucrose per liter). Every strawberry fruit was maintained in this medium for all assay period (6 days), with new fresh medium changes, every two days. A modified protocol of agroinfiltration previously described (Spolaore et al., 2001; Hoffmann et al., 2006) was performed to reduce variability among fruits and be able to compare the defense response to pathogen inoculations between halves of the same fruit (Supplementary Figure S1). Thus, a half of the fruit was infiltrated with *Agrobacterium* bearing the query transgene construct and the opposite half with *Agrobacterium* bearing the corresponding empty vector, as a control. In order to clearly distinguish both fruit halves for later manipulations, two sepals were removed from the half corresponding to the query constructs (either silencing or overexpression) before agroinfiltration. Short needles (23Gx25 mm) were employed for infiltration to ensure that each *Agrobacterium* suspension remains in the corresponding fruit half and did not spread within the opposite half of fruit. The agroinfiltration was carried out in the center of every strawberry fruit half, approximately. Although 1 ml of *Agrobacterium* suspension was infiltrated in most of strawberry fruit halves, this volume was slightly adjusted according to the size of the fruit until complete run off.

Two days after the agroinfiltration, both halves of the fruit were inoculated with *C. acutatum* using 5 mm paper discs embedded in a 10^5^ conidia/mL suspension. The embedded discs were placed on the strawberry surface mainly located on the agroinfiltration point. A subset of the agroinfiltrated fruits was reserved as “non-infected fruits” and was not inoculated with the pathogen. All fruits were stored in closed chamber with 75–80% humidity at 25°C for five days. Three fruits were collected every day and samples from each of the two halves of the collected fruits were immediately frozen in liquid nitrogen and transferred to -80°C until use.

Healthy fruits grown under field conditions were selected and used following our experimental design, which was two times repeated during strawberry fruiting season for two years (288 fruits for the silencing or overexpression, including its corresponding control). The total number (576) of strawberry fruits used in this work is summarized in Supplementary Table S2. For silencing of *FaWRKY1* gene, 144 out of 288 fruits were agroinfiltrated with the pFRN::FaWRKY1-RNAi construct in one fruit half and with the corresponding empty vector in the opposite fruit half. To analyze the biological effect of the agroinfiltration process in the expression of the endogenous *FaWRKY1* gene, 144 fruits were agroinfiltrated only in one fruit half with the empty vector but no agroinfiltration was accomplished in the corresponding opposite fruit half. In every case, 120 out of the 144 fruits were inoculated with the pathogen in both fruit halves (24 out of 120 fruits were used for expression studies and 96 remaining fruits for statistical purposes), leaving 24 fruits without inoculation as control, in order to analyze the silencing of *FaWRKY1* upon no infection condition. From each experimental condition, three fruits were collected every 24 h for 6 days, which were used for the *FaWRKY1* gene expression analysis by RT-qPCR. A similar number of fruits and identical protocol was followed for the *FaWRKY1* overexpression experiments but plasmid 35S::FaWRKY1 was used instead.

### Histochemical GUS Assay

A total of 30 strawberry transient transform fruit halves (three fruits were collected every 24 h for 7 days) were used for the histochemical assay. GUS activities in strawberry was performed as described by Jefferson et al. (1987) using a modified staining solution following the manufacturer (Gold Biotechnology) instructions containing: 2 mM X-gluc in 100 mM sodium phosphate buffer (pH 7.5), 10 mM EDTA, 0.1% (v/v) Triton X-100, 1.0 mM potassium ferricyanide.

### Total RNA Extraction and Real-Time qPCR

Total RNA from strawberry tissues was isolated as described previously (Casado-Díaz et al., 2006), treated with DnaseI (Invitrogen) for residual DNA removal, and further purified with the RNeasy MinElute Cleanup Kit (QIAGEN). Purified RNA was quantified by NanoDrop 1000 Spectrophotometer (Thermo scientific). RNA integrity was checked using the Agilent 2100 Bioanalyzer (Agilent Technologies, Germany). First-strand cDNA synthesis was carried out using 1 μg of purified total RNA as template for a 20 μL reaction [iScript cDNA Synthesis kit (Bio-Rad)]. RT reactions were diluted 5-fold with nuclease-free water prior to RT-qPCR.

Specific primer pairs set were designed using Oligo Primer Analysis software version 6.65, tested by dissociation curve analysis, and verified for absence of non-specific amplification. The expression levels were calculated according to the 2^−ΔΔCT^ method (Livak and Schmittgen, 2001) and, normalized according to two housekeeping gene *actine 1* (*FaACT1)* and *elongation factor 1α* (*FaEF1a)* (Amil-Ruiz et al., 2013). RT-qPCR runs were performed using specific primers (Supplementary Table S1) in two technical replicates in the same run and three biological replicates in different runs, as described previously (Encinas-Villarejo et al., 2009), using SsoAdvanced^TM^ SYBR^®^ Green Supermix, and MyIQ v1.004 and iCycler v3.1 real-time PCR systems (Bio-Rad). Mean PCR efficiencies were calculated by LinRegPCR software (Ruijter et al., 2009). All RT-qPCR primers used in this study have similar PCR efficiencies.

The level of silencing and overexpression of *FaWRKY1* was calculated for each time and normalized as the relative expression value of this gene between the agroinfiltrated fruit half with the query cassette construct and the corresponding agroinfiltrated opposite fruit half with the control vector.

### Tissue Fruit Damage Evaluation and Statistical Analysis

Seventy fruits (*n* = 70) for the silencing and sixty fruit (*n* = 60) for the overexpression experiment were phenotypically observed and evaluated for tissue damage at 4 days post inoculation with *C. acutatum*. The severity of tissue damage was carried out on 5-scale according to (Choi et al., 2016). Essentially, **1**, symptomless tissues (0% fruit halve damaged); **2**, weakly visible lesion (up to 10% fruit halve damaged); **3,** moderate lesion (10–25% fruit halve damaged); **4,** enlarged lesion (25–50% fruit halve damaged); **5**, very affected fruit (> 50% fruit halve damaged). Two different ratios were calculated: internal damage ratio and external damage ratio, both resulting from dividing the internal or external tissue damage value of the fruit half where the transgene was overexpressed or silenced by the tissue damage value corresponding to the opposite half of the same fruit infiltrated with *Agrobacterium* bearing the empty vector. Fruits where both halves were infiltrated with Agrobacterium bearing the empty vector were used as control for statistical purposes. Means and SE were obtained by Fisher’s LSD test (α = 0.05) by Statistix software (v9.0). A ratio of 1, clearly indicate no differences between both halves of the same fruit.

Real Time-qPCR data were statistically analyzed in Microsoft Excel, using the Real Statistics Resource Pack software, release 5.4^2^. All data were tested for normality using Shapiro-Wilk test (α = 0.05). One-way ANOVA, followed by Dunnett’s test or Tukey’s test *post hoc* were performed at α = 0.05 and 0.01. Three biological replicates were used (*n* = 3).

## Results

### Spatial-Temporal Expression Analyses of the Transgene After *Agrobacterium* Infiltration in Strawberry Fruits

**FIGURE 1:**
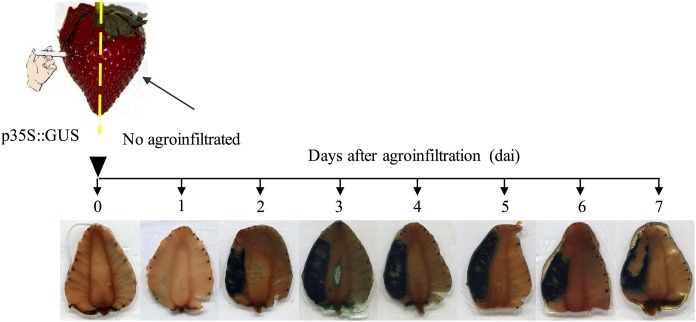
Spatial and time course expression analysis of GUS reporter gene in strawberry fruit (*Fragaria* × *ananassa*) agroinfiltrated with pCaMV35s::GUS-GFP (p35S::GUS). Histochemical GUS staining was performed in longitudinal sections of fruits which only one half of the fruit was agroinfiltrated with *A. tumefaciens* carrying the plasmid pCaMV35s::GUS-GFP. The GUS activity was determined every 24 h, up to seven days after agroinfiltration, as described in materials and methods. The agroinfiltrated fruit half was marked and easily distinguished by removing two sepals before infiltration and it is shown as the left side of every fruit slice in this picture.

To validate the methodology and to identify the spatio-temporal gene expression of the transgene after strawberry fruit *Agrobacterium* infiltration (agroinfiltration) in our experimental conditions, β-glucuronidase (GUS) activity was determined in fruits in which only one half was agroinfiltrated with the construct p35S::GUS (Figure 1). GUS activity was monitored in longitudinal sections of these strawberry fruits every 24 h, up to seven days after agroinfiltration (7 dai). As shown in Figure 1, GUS activity became clearly visible at the second day after agroinfiltration (2 dai), and this expression was detected only within the agroinfiltrated fruit half. Interestingly, GUS activity slightly increased at 3 dai and it was maintained up to 7 dai and was confined only within the agroinfiltrated fruit half being limited by the pith. Therefore, no activity was clearly visible in the opposite half of fruit and aloof regions of injection point after seven days (Figure 1).

Considering these data, two days after agroinfiltration (2 dai) was chosen as the appropriate time to make the *C. acutatum* inoculations in those experiments designed to test loss and gain of *FaWRKY1* function by *Agrobacterium* transient expression in fruit.

### Changes in the Expression Pattern of *FaWRKY1* After *Agrobacterium* Infiltration and *C. acutatum* Inoculation Are Independently Distinguished in Strawberry Fruit

**FIGURE 2:**
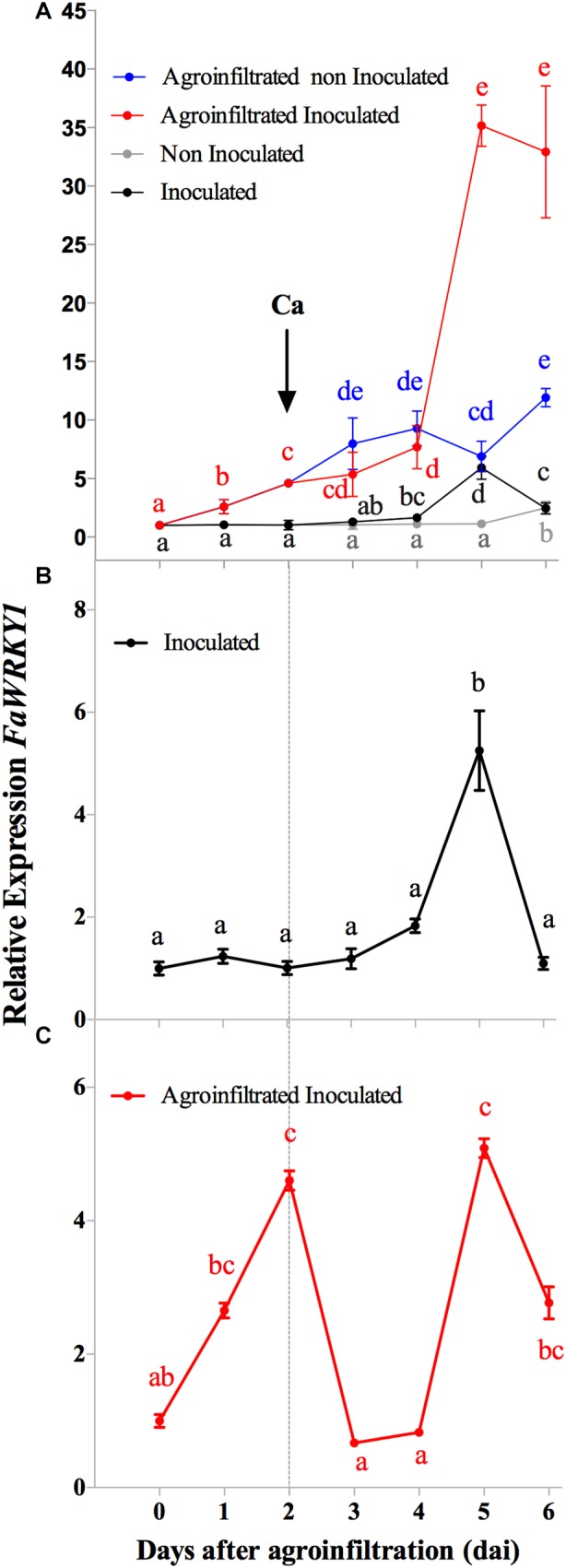
Gene expression pattern of *FaWRKY1* in strawberry fruit after *A. tumefaciens* infiltration and *C. acutatum* inoculation. To easy follow, time scale is represented here along 6 days after fruit infiltration with *A. tumefaciens* bearing the pFRN vector even though in panels **A,B**, non-agroinfiltrated fruit are represented. The arrows indicate the time of inoculation with *C. acutatum* (Ca). In panels **A–C**, the relative expression of *FaWRKY1* is represented with respect to time zero; **(A)** relative expression of *FaWRKY1* in agroinfiltrated or non-agroinfiltrated strawberry fruit upon *C. acutatum* infection or mock; **(B)** the relative expression of *FaWRKY1* in non-agroinfiltrated and inoculated fruit was normalized with respect to the non-agroinfiltrated non-inoculated ones; **(C)**, the *FaWRKY1* expression in pFRN agroinfiltrated and inoculated fruit was normalized with respect to the agroinfiltrated and non-inoculated ones. In the graphics, standard value 1 at T0 was added to better illustrate changes. Values are the means of three biological replicates. Means followed by the same letters in each trait are not significantly different (α = 0.05), according to Tukey’s test.

A time course analysis by real-time PCR was performed on strawberry fruits after agroinfiltration and *C. acutatum* inoculation. Thus, fruits were agroinfiltrated in one half with the empty pFRN vector. Two days after agroinfiltration, 24 out of the 48 fruits were also inoculated in both halves with *C. acutatum*, while the other 24 fruits remained uninfected (treated with mock-soaked discs), as control reference. In order to distinguish changes in the *FaWRKY1* expression pattern only due to a response of the fruit to *A. tumefaciens* infiltration and/or *C. acutatum* infection, the expression pattern of *FaWRKY1* was determined over time by comparing both, fruit halves agroinfiltrated and inoculated *versus* fruit halves agroinfiltrated and non-inoculated, and fruit halves only inoculated *versus* non-inoculated ones (Figure 2).

Results in Figure 2A show the complexity of the *FaWRKY1* expression pattern under all conditions tested. When data from fruits inoculated with *C. acutatum* was normalized to non-inoculated ones, a significant accumulation of *FaWRKY1* transcripts were detected in response to this pathogen inoculation, which reached a peak level at 5 dai, corresponding to 3 days post inoculation (3 dpi) (Figure 2B). This expression decreased to lower level at 6 dai (4 dpi). Moreover, the expression pattern of *FaWRKY1* was more complex when data from agroinfiltrated and inoculated fruits was normalized to the corresponding agroinfiltrated and non-inoculated ones (Figure 2C). Thus, *FaWRKY1* was significantly induced in response to *Agrobacterium* infiltration (Figure 2C) and this expression reached its peak at 2 days after infiltration (2 dai), but decreased to lower levels at 3 dai. In addition, a fast increase in *FaWRKY1* transcript accumulation was later detected, reaching peak levels at 5 dai and quickly decreasing at 6 dai. Interestingly, this second peak of expression correlated to that showed in Figure 2B, thus indicating that *C. acutatum* was able to induce *FaWRKY1* expression in the agroinfiltrated fruits in a similar way to that observed in the non-agroinfiltrated fruits. These results state the expression pattern of *FaWRKY1* gene in strawberry fruit in our experimental system after *Agrobacterium* infiltration and *C. acutatum* inoculation and indicate that changes in the expression pattern of this gene due to each event are independently distributed and can be distinguished over time (Figure 2C).

### The Transient Silencing of *FaWRKY1* Gene in Strawberry Fruit Reduced Fruit Tissue Damage After *C. acutatum* Inoculation

Strawberry fruits were independently agroinfiltrated on opposite halves of the same fruit with silencing (pFRN::FaWRKY1-RNAi) and empty vector (pFRN) constructs. Two days after agroinfiltration, 24 out of the 48 fruits were also inoculated in both halves with *C. acutatum*, while the other 24 fruits remained uninfected (treated with mock-soaked discs) (see experimental design details in Supplementary Figure S1).

Changes in the expression pattern of *FaWRKY1* were analyzed in agroinfiltrated fruit halves, which were either not exposed (Supplementary Figure S2A) or exposed (Supplementary Figure S2B) to *C. acutat*um infection. Results in Supplementary Figure S2A show the induction pattern of the *FaWRKY1* gene over time, due to the agroinfiltration event in both pFRN and pFRN::FaWRKY1-RNAi agroinfiltrated fruit halves. This induction was always much lower in pFRN::FaWRKY1-RNAi silenced fruit halves compared to that of pFRN ones. Increases in *FaWRKY1* transcript accumulation after *C. acutatum* inoculation were also detected in fruit halves which were previously and independently agroinfiltrated with empty vector (pFRN) and pFRN::FaWRKY1-RNAi silencing construct, respectively (Supplementary Figure S2B). Also, a much lower level of *FaWRKY1* expression was detected along all time points for pFRN::FaWRKY1-RNAi fruit halves compared to pFRN ones. This reduction in the *FaWRKY1* expression was markedly relevant at 5 dai (corresponding to 3 dpi), where an increasing value of 35-fold was detected for the expression of *FaWRKY1* gene in pFRN fruit halves whereas less than 9-fold increase was only detected in fruit halves agroinfiltrated with pFRN::FaWRKY1-RNAi.

The level of *FaWRKY1* gene silencing was calculated for every time and condition by normalizing the silenced fruit halves values to the control ones of the corresponding opposite fruit halves, and it is also represented in Supplementary Figures S2A,B. A reduction of *FaWRKY1* transcript accumulation was found as early as two days after agroinfiltration and remarkable silencing values were observed at 4 dai (2 dpi), 5 dai (3 dpi), and 6 dai (4 dpi) in both inoculated and non-inoculated fruit.

These results clearly indicated that in our experimental system the *FaWRKY1* gene is successfully silenced in strawberry fruit after agroinfiltration with the pFRN::FaWRKY1-RNAi silencing construct.

**FIGURE 3:**
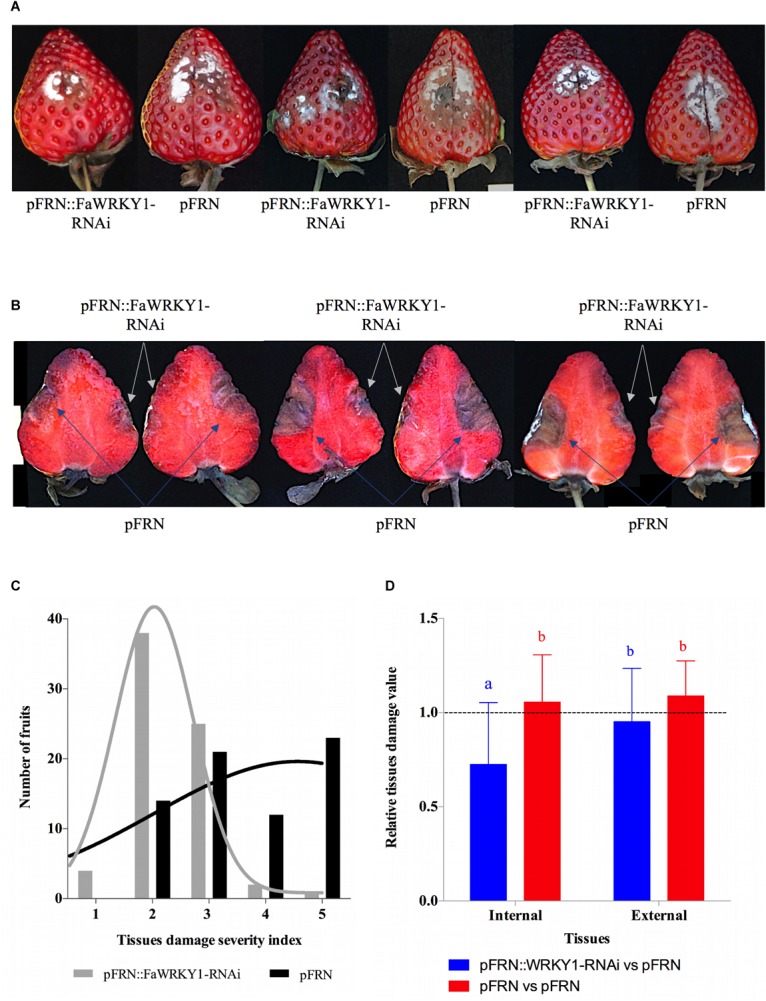
The silencing of *FaWRKY1* in strawberry fruit enhances resistance to *C. acutatum* infection. **(A)** External surface disease symptoms on the two agroinfiltrated opposite halves (pFRN::FaWRKY1-RNAi and pFRN) of the same fruit. Three different fruits are shown as an example. **(B)** Internal tissue damage, of the same three fruits shown in panel **A**; white and blue arrows indicated the tissue area affected in the pFRN::FaWRKY1-RNAi or pFRN empty vector agroinfiltrated fruit half, respectively, at 4 dpi. **(C)** Distribution of a total of 70 strawberry fruits based on a 1 to 5 scale used to asses tissue damage in each of the two opposite fruit halves of the same fruit (1, no symptoms; 2, weakly visible lesion; 3, moderate lesion; 4, enlarged lesions; 5, very affected); black and gray bars, number of pFRN and pFRN::FaWRKY1-RNAi agroinfiltrated fruit halves, respectively, exhibiting the indicated grade of tissue damage at 4 dpi with *C. acutatum*; black and gray lines, indicate the distribution of each fruit half according to the severity scale. **(D)** Statistically analysis of internal and external tissue damage ratio of the two opposite halves of the same fruit, according to the 1 to 5 severity scale; blue and red bars, pFRN::WRKY1-RNAi/pFRN and pFRN/pFRN agroinfiltrated values, respectively. Data correspond to mean ± SD. Within each bars, means with different letters are significantly different by LSD test at *p* < 0.05. A ratio value of 1, clearly indicate no differences between opposite halves of the same fruit.

Taking into account all these results, the evaluation of fruit tissue damage and the comparative analysis of susceptibility to *C. acutatum* between the two halves of the same fruit (one agroinfiltrated with the silencing construct and the other with the empty vector as control) were accomplished after 6 dai (4 dpi), in a total of 70 fruits (Figure 3). In general, no relevant differences in the external tissue damage were visually observed in both opposite halves of the same fruit. Thus, mycelial growth, accompanied by tissue browning and depressed necrosis, was clearly visible surrounding the inoculation area after 4 dpi (Figure 3A). However, when the internal tissue damage was evaluated, a relevant reduction was clearly detected within fruit halves agroinfiltrated with the silencing construct compared to the corresponding opposite fruit halves agroinfiltrated with the empty pFRN vector (Figure 3B).

Also, the distribution of damaged fruit, based on a 1 to 5 scale, was evaluated (Figure 3C). Hence, approximately 60% of the pFRN::FaWRKY1-RNAi agroinfiltrated fruit halves presented none or very small tissue damage (scores 1, and 2), while 36% showed moderate damage (score 3), and only 4% presented a very large affected tissue area (scores 4, and 5). On the other hand, only 20 and 30% of the pFRN agroinfiltrated fruit halves showed small (score 2) or moderate (score 3) tissue damage, respectively, while a higher percentage of up to 50% were strongly affected by *C. acutatum* infection (scores 4, and 5) (Figure 3C).

A statistical analysis of the internal and external fruit tissue damage was conducted and is shown in Figure 3D. For internal fruit tissue damage, the values obtained by normalizing fruit halves transformed with the silencing construct with respect to the corresponding opposite fruit halves transformed with the empty pFRN vector were significantly reduced (mean value of 0,7257) compared to those obtained when both fruit halves were transformed with pFRN control constructs (mean value 1,0556). On the contrary, for external fruit tissue damage, all the ratio values show no significant differences.

These results establish a positive correlation between the silencing of the *FaWRKY1* gene and an increase of fruit resistance to *C. acutatum* (Figure 3D).

### The Transient Overexpression of *FaWRKY1* in Strawberry Fruit Did Not Alter Susceptibility to *C. acutatum*

The expression pattern of *FaWRKY1* gene was analyzed in inoculated fruits in which one half was agroinfiltrated with the 35S::FaWRKY1 overexpression construct, and the opposite half with the empty pK7WG2 vector as control (Supplementary Figure S3). Based on the results observed in Supplementary Figure S2 for the silencing experiment, the expression of this transgene was expected to be highly induced at 4 and 5 dai. Accordingly, the *FaWRKY1* gene was highly induced, at both the fourth and fifth day after agroinfiltration (Supplementary Figure S3) in fruit halves transformed with the 35S::FaWRKY1 construct, in comparison to that of the corresponding opposites fruit halves both transformed with the pK7WG2 control vector.

The level of *FaWRKY1* overexpression was also calculated for every time by normalizing the overexpressed fruit halves values to that of the control ones of the corresponding opposite fruit halves, and it is also represented in Supplementary Figure S3 (red line). Thus, increases of *FaWRKY1* transcript level of 20-fold and 10-fold that of control were found at 4 dai and 5 dai, respectively, in fruit halves transformed with the overexpression vector.

These results clearly indicated that in our experimental system the *FaWRKY1* gene is successfully overexpressed in strawberry fruit after agroinfiltration with the 35S::FaWRKY1 construct.

**FIGURE 4:**
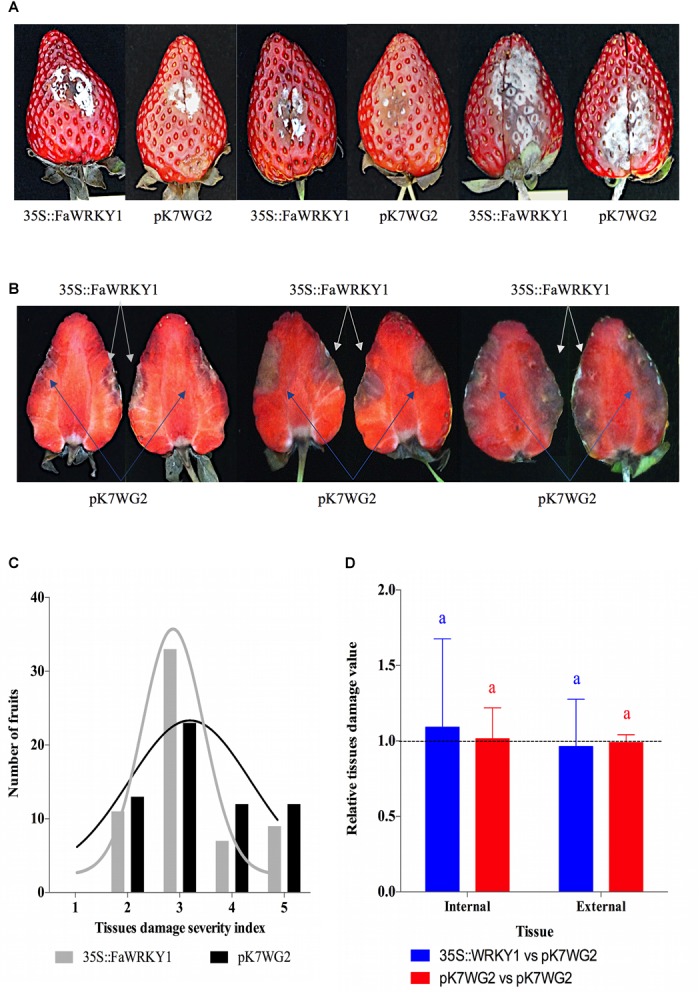
Transient overexpression of *FaWRKY1*-gene in strawberry fruit does not substantially alter susceptibility to *C. acutatum* infection. **(A)** External surface disease symptoms on the two agroinfiltrated opposite halves (pK7WG2::FaWRKY1 and pK7WG2.0) of the same fruit. Three different fruits are shown as an example. **(B)** Internal tissue damage of the same three fruits shown in panel **A**; white and blue arrows indicates the tissue area affected in the pK7WG2::FaWRKY1 (35S::FaWRKY1) or pK7WG2.0 empty vector agroinfiltrated fruit half, respectively, at 4 dpi. **(C)** Distribution of a total of 60 strawberry fruits based on a 1 to 5 used to asses tissue damage in each of the two opposite fruit halves of the same fruit (1, no symptoms; 2, weakly visible lesion; 3, moderate lesion; 4, enlarged lesions; 5, very affected); black and gray bars, number of pK7WG2.0 and pK7WG2::FaWRKY1 agroinfiltrated fruit halves, respectively, exhibiting the indicated grade of tissue damage at 4 dpi with *C. acutatum*; black and gray lines, indicate the distribution of each fruit halves according to the severity scale. **(D)** Statistical analysis of internal and external tissue damage ratio of the two opposite halves of the same fruit, according to the 1 to 5 severity scale; blue and red bars, pK7WG2::FaWRKY1/pK7WG2.0 and pK7WG2.0/pK7WG2.0 values, respectively. Data correspond to mean ± SD. Within each bars, means with different letters are significantly different by LSD test at *p* < 0.05. A ratio value of 1, clearly indicate no differences between opposite halves of the same fruit.

The evaluation of fruit tissue damage and the comparative analysis of susceptibility to *C. acutatum* between the two opposite halves of the same fruit (one agroinfiltrated with the 35s::FaWRKY1 construct and the other with the pK7GW2.0 empty vector as control) were accomplished after 6 dai (4 dpi), in a total of 60 fruits (Figure 4), as previously described for the silencing experiments. Again, no relevant differences in the external tissue damage were visually observed between opposite halves (Figure 4A). When the internal tissue damage was evaluated, no relevant differences were also visually detected between fruit halves transformed with the 35S::FaWRKY1 construct compared to the corresponding opposite fruit halves transformed with the empty pK7GW2.0 vector (Figure 4B). In addition, the distribution pattern of damaged fruit was similar for the two fruit halves, irrespectively of the agroinfiltrated construct (Figure 4C). Interestingly, damage-free fruit halves were not observed and only few fruits (10 and 12% of the overexpressed and control fruit halves, respectively) showed small damaged region (score 2). Instead, most of fruit halves showed moderated damage (score 3), and a similar high percentage of fruit showed large or very large tissue damage (scores 4 and 5) on both fruit halves. In fact, the statistical analysis of internal and external fruit tissue damage did not show any significant difference, irrespectively of the agroinfiltrated construct (Figure 4D).

These results indicate that the transient overexpression of *FaWRKY1* in strawberry fruit does not seem to substantially affect susceptibility to *C. acutatum.*

### Expression Pattern of Potential FaWRKY1 Target Genes in Strawberry After *C. acutatum* Inoculation

**FIGURE 5:**
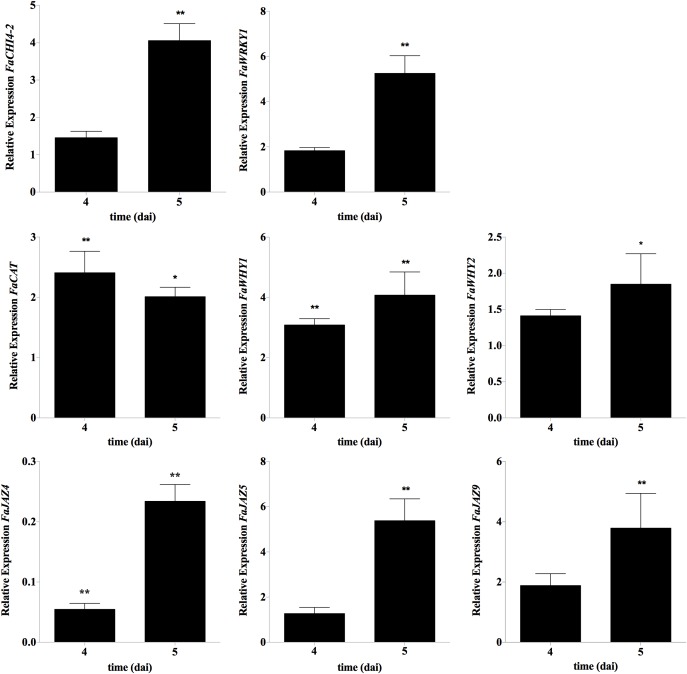
Gene expression patterns of potential *FaWRKY1*-responsive strawberry genes, after *C. acutatum* inoculation. RT-qPCR analysis was accomplished in non-agroinfiltrated strawberry fruits. The time scale is shown as for agroinfiltration fruit samples (4 and 5 dai) even though the expression analyses was carried out at this time points in non-agroinfiltrated strawberry fruits (corresponding to 2 and 3 days post inoculation with *C. acutatum*). Relative expression as the fold change between the inoculated vs. non-inoculated fruit was calculated by the 2^−ΔΔCT^ method. *FaCHI4-2* and *FaWRKY1* were included as positive controls. Mean, standard error and significant differences found by Dunnett’s test are represented (^∗^*p* ≤ 0.05, ^∗∗^*p* ≤ 0.01; *n* = 3).

In order to uncover downstream FaWRKY1 defense responsive elements in strawberry, the expression of some strawberry orthologs of genes previously described in *Arabidopsis* as WRKY75 target genes (Supplementary Table S1) was evaluated after *C. acutatum* inoculation in non-agroinfiltrated fruit and in both *FaWRKY1* silenced and overexpressed fruit, at 4 and 5 days of agroinfiltration, where the highest levels of *FaWRKY1* transient silencing and overexpression were detected (Supplementary Figures S2, S3). Only *FaCAT*, *FaWHY1*, *FaWHY2*, *FaJAZ9*, and *FaJAZ5* genes responded positively to *C. acutatum* infection, and were significantly upregulated at 2 dpi (4 dai) and/or 3 dpi (5 dai) in non-agroinfiltrated fruit, being the expression of *FaJAZ4* significantly down-regulated at both times point (Figure 5). No significant changes in gene expression were observed for *FaJAZ1*, *FaJAZ8.1*, *FaJAZ10*, and *FaJAZ12* and *FaICS1* neither at 2 dpi (4 dai) nor 3 dpi (5 dai) (data not shown). Also, no significant change in the expression of any of the strawberry orthologs was detected at 2 and 3 dpi, when *FaWRKY1* was transiently silenced or overexpressed (data not shown).

## Discussion

To get insight into the role of *FaWRKY1* in the strawberry defense response to pathogens, we have accomplished the transient silencing and overexpression of this gene in strawberry fruits by *Agrobacterium* mediated transformation, which were later analyzed upon *C. acutatum* inoculation. Transient expression by agroinfiltration has been applied to strawberry fruit as an efficient system to characterize genes associated with fruit development, physiology and metabolism (Carvalho et al., 2016). However, this methodology presents the limitation that *Agrobacterium* itself is an unusual plant pathogen, which can hamper the study of other plant-pathogen interactions (Guidarelli and Baraldi, 2015; Carvalho et al., 2016). Here, a modified experimental system where opposite halves of the same fruit are transiently and independently transformed has allowed the study of the effect of a transgene and its control in the same single fruit so that gene expression was only confined within the tissue area of the corresponding injected fruit half (Figure 1, 2 and Supplementary Figure S1). This fact is particular interesting since to date, the process of infiltration has been carried out by a single injection of the *Agrobacterium* culture into the whole fruit, with a uniform expression of the transgene both close and distant from the injection sites (Hoffmann et al., 2006; Guidarelli et al., 2014). An advantage of using this technique is that the effect of a transgene produced in one half of the fruit can be normalized with respect to the control vector, in the opposite half (Supplementary Figure S2, red scale) and variability is strongly reduced, as the physiological/developmental stages between opposite fruit halves are identical or closely identical.

### The FaWRKY1 Negatively Regulates Resistance to *C. acutatum* in Strawberry Fruit Upon Infection

*FaWRKY1* gene expression was efficiently silenced after infiltration with *Agrobacterium* bearing the silencing cassette construct in both *C. acutatum* inoculated and non-inoculated fruit halves (Supplementary Figure S2). As the level of silencing of *FaWRKY1* was relevant at 2 days after agroinfiltration (25% of gene silencing) in non-inoculated fruit, this time point was selected to inoculate *C. acutatum* for further fruit susceptibility assays, also bearing in mind that this pathogen takes around 24 h to develop subcuticular intracellular hyphae (Guidarelli et al., 2011; Amil-Ruiz et al., 2016). In this way, we matched the initial phases of growth and development of the fungi with the time when a very high value of *FaWRKY1* silencing was detected in agroinfiltrated fruit.

The fruit susceptibility assays demonstrated that down-regulation of *FaWRKY1* gene in fruit enhances resistance to *C. acutatum* (Figure 3). Thus, tissue damage was visually reduced in fruit halves that were infiltrated with *Agrobacterium* bearing the silencing construct compared to that of the opposite control halves (Figure 3B). Also, most of the silenced fruit halves scored lower values of tissue damage (mainly 1 to 3) than control fruit halves, which grouped at higher values of severity (scores 3 to 5) (Figure 3C). Furthermore, when tissue damage values were normalized between opposite halves, enhanced resistance was found statistically significant in the silenced fruit (Figure 3D). Taking together, these results evidence a role of FaWRKY1 in strawberry fruit as negative regulator of resistance to the pathogen *C. acutatum*. It is worthwhile to note that no clear difference in external surface damage was detected between silenced and control fruit. Although alternative explanations are plausible, it can presumably be due to a much lower transformation event produced in surface fruit cells than in internal tissues cells as a consequence of the agroinfiltration procedure.

### Ectopic Expression of *FaWRKY1* in Strawberry Fruit Does Not Increase Susceptibility to *C. acutatum*

The transient overexpression of *FaWRKY1* gene in response to *C. acutatum* did not result in any change in fruit susceptibility to this pathogen (Figure 4) even though a clear and significant accumulation of *FaWRKY1* transcripts was detected at 4 and 5 days after agroinfiltration (Supplementary Figure S3). Thus, neither external nor internal clear distinguishing morphological differences were visually observed between fruit halves infiltrated with *Agrobacterium* bearing the overexpression cassette and the corresponding opposite fruit halves bearing the empty vector, as control (Figure 4A,B). The distribution pattern of fruit halves according to their tissue damage was also similar for both overexpressed and control fruit halves (Figure 4C) and no significant differences were found when tissue damage values were normalized between the two opposite halves of the same fruit (Figure 4D).

Although increase of fruit susceptibility after *FaWRKY1* overexpression could be expected in fruit cells, however, the presence of high levels of this transcription factor acting as a gene repressor may not necessarily end in increased susceptibility to this pathogen. In normal uninfected conditions, a basal *FaWRKY1* expression in plant cells can be enough to have the complete set of specific FaWRKY1-responsive defense genes repressed in order to optimize plant fitness (Huot et al., 2014). Thus, high levels of FaWRKY1 proteins present in fruit cells after *FaWRKY1* ectopic expression would not add more repression on these FaWRKY1-related genes but this repression would remain unaltered. Previous studies in *Arabidopsis* had revealed a repressor role of FaWRKY1 on defense related genes when this *FaWRKY1* gene was ectopically overexpressed in wild type plants (Encinas-Villarejo et al., 2009). However, in that study, the overexpression of strawberry *FaWRKY1* gene in *Arabidopsis* Atwrky75 mutant, restored the susceptible phenotype to wild-type and even increased resistance of the mutant to avirulent strains of *P. syringae*, (Encinas-Villarejo et al., 2009). A similar pattern of pathogen resistant has also been described by Guo et al. (2017) in *Arabidopsis WRKY75ox* (over-expressing) lines, which show an elevated SA content and enhanced resistance to *Pst* DC3000, compared with both, wild type Col-0 and *WRKY75RNAi* plants.

What are the molecular events underlying a different plant defense response after the ectopic expression of *FaWRKY1* in strawberry and *Arabidopsis* remain to be further elucidated. Multiple function variability and positive or negative regulator roles have been described for AtWRKY75 and its homologs in other species. Interestingly, high levels of gene expression were detected for the WRKY75-like orthologous gene in a peach resistant cultivar to *Xanthomonas arboricola* compared with a more susceptible cultivar (Gervasi et al., 2018). Also, overexpression of *VvWRKY1* (AtWRKY75-like) in grapevines enhanced resistance to biotrophic *Plasmopara viticola*, the causal agent of downy mildew, through induction of JA-pathway related genes (Marchive et al., 2013). Contrarily, and accordingly to our results in the present report, the silencing of *GbWRKY1* in cotton enhanced plant resistance to hemibiotrophic fungal *V. dahliae and B. cinerea* (Marchive et al., 2013; Li et al., 2014).

Taken together all these results, one might speculate on WRKY75-like TFs acting as a positive regulator of resistance against both bacterial and biotrophic fungi pathogens, whereas it negatively regulates defense responses against fungi with a necrotrophic phase or lifestyles. However, it is also worthwhile to note that the positive regulator role of FaWRKY1 in pathogen resistance observed in *Arabidopsis* was detected in tissue plant other than fruit. It is known that hormones modulate plant immunity, with SA and JA as major players. However, ET, ABA, GA, auxins, cytokinins, brassinosteroids and nitric oxide, also have pivotal roles in the regulation of the plant immune signaling network (Pieterse et al., 2012). Moreover, interplay between phytohormones is required for development, maturation, and ripening of fruit (Mcatee et al., 2013) and thus, strawberry fruit tissue undertakes substantial changes in the hormonal balance over growing and ripening time (Medina-Puche et al., 2016), which could affect main defense pathways differently from other tissues.

Together, our results in strawberry indicate that the overexpression of *FaWRKY1* in fruit does not substantially affect fruit susceptibility to *C. acutatum*, whereas strong evidence is also provided that the silencing of *FaWRKY1* in strawberry fruit enhances resistance to *C. acutatum*. Also, these results evidence the complexity and multiple layers of control that FaWRKY1 can exhibit and highlight differences in the defense response strategies activated either by AtWRKY75 or FaWRKY1 proteins according to different plant species, plant tissue and/or different style of life deployed by *P. syringae* or *C. acutatum* pathogens, respectively.

### Downstream Defense Responsive Elements and Underlying Mechanisms of WRKY75-Like Genes and FaWRKY1 in Strawberry

As mentioned before, and accordingly to our results in strawberry, the silencing of *GbWRKY1* in cotton has been reported to enhance plant resistance to hemibiotrophic fungal *V. dahliae and B. cinerea* (Marchive et al., 2013; Li et al., 2014). Interestingly, GbWRKY1 acted by promoting transcription of *JAZ1* homologs. JA ZIM-domain (JAZ) family proteins are repressors interacting with several transcriptional factors involved in the regulation of early JA-responsive genes (Chini et al., 2007; Fernandez-Calvo et al., 2011; Major et al., 2017). Thus, at low JA-Ile levels, JAZ protein negatively regulates JA signaling pathway, including many TFs which positively regulate JA-responsive genes (Gimenez-Ibanez et al., 2016), whereas in presence of active form of JA (JA-Ile), JAZ proteins are targeted by the SCF^COI1^ complex, and are subsequently degraded by the 26S proteasome (Chini et al., 2007; Thines et al., 2007; Kazan and Manners, 2012).

On the other hand, WRKY75 positively regulates plant resistance to *P. syringae* in *Arabidopsis*, but is also upregulated during leaf senescence, a complicated process influenced by a large number of genes, environment, stresses and endogenous levels of phytohormones (Guo et al., 2017; Li et al., 2017). This senescence process is regulated by WRKY75, which promotes the SA biosynthesis, directly activating the transcription of *Isochorismate synthase1* (*ICS1* or *SID2*), and H_2_O_2_ accumulation (Guo et al., 2017; Li et al., 2017). Interestingly, *FaWRKY1* is also up-regulated in over ripen strawberry fruit (Encinas-Villarejo et al., 2009). Also, AtWRKY75 is able to suppress catalase activity by directly repressing CAT2 transcription, which directly contributes to increase the production of ROS (Guo et al., 2017; Li et al., 2017). High levels of ROS strongly correlate with the induction and maintenance of the cell senescence process and the HR, as also described elsewhere (Jajic et al., 2015). Furthermore, positive regulation of the defense response has been found recently involving the cassava WRKY75 homolog (MeWRKY75) interacting with WHY TF (Liu et al., 2018). MeWRKY75 positively regulates disease resistance to cassava bacterial blight first activating the expression of *MeWHY3* gene through directly binding to the W-box of its promoter region, and then promoting its physical interaction with MeWHYs. The physical interaction between MeWHYs and MeWRKY75 contributes to the activation of defense-related genes and improves resistance against the biotrophic *Xanthomonas axonopodis* (Liu et al., 2018). Interestingly, AtWHYs are also involved in modulating leaf senescence (Miao et al., 2013; Ren et al., 2017).

**FIGURE 6:**
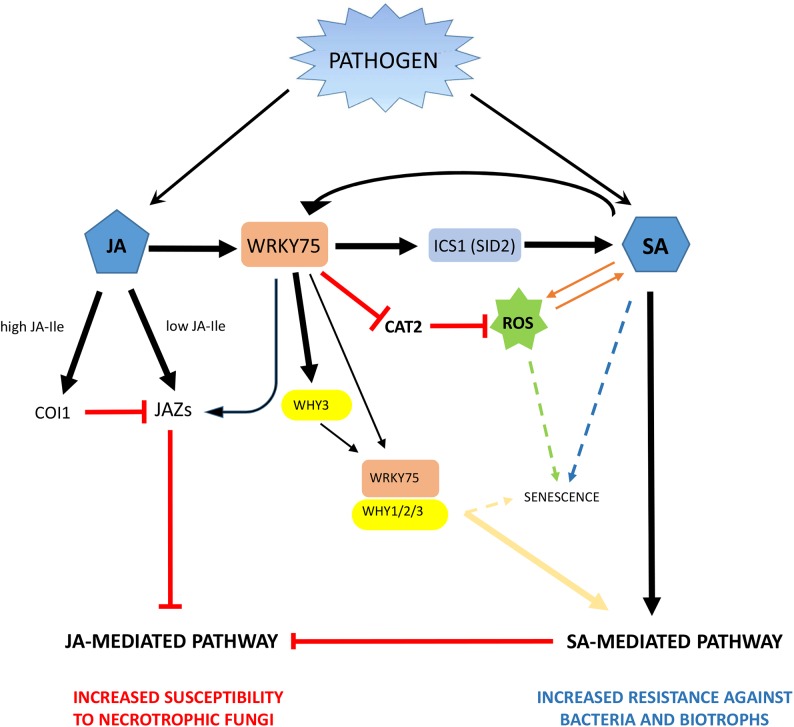
Schematic of a tentative model for downstream regulatory networks dictated by AtWRKY75-like genes in plants. The model is based on the results described for AtWRKY75 and its homologs in green tissue from species other than strawberry. Thus, the cotton GbWRKY1 (AtWRKY75-like) can activate JAZ1 expression, which interferes with the JA-mediated defense pathway and negatively regulate plant resistance to the pathogens *B. cinerea* and *V. dahliae* (Li et al., 2014). In addition, AtWRKY75 directly bind to the Isochorismate synthase1 (SID2) promoter to positively regulate its transcription and stimulate the biosynthesis of SA, which in turn seems to promote the activation of SA-mediated pathway and the generation of reactive oxygen species (ROS) and positively regulate plant resistance to *P. syringae* (Guo et al., 2017). Also, AtWRKY75 is able to suppress catalase activity by directly repressing CAT2 transcription, which directly contributes to increase the production of ROS (Guo et al., 2017). Other interactions of WRKY75 homologs, includes WHIRLY factors, as described for the cassava MeWRKY75 (Liu et al., 2018). Thus, MeWRKY75 is able to activate MeWHY3 transcription and also physically interact with WHY factors to form a protein complex and mediate disease resistance to cassava bacterial blight infection (Liu et al., 2018). Interestingly, WHY1 has also been associated to senescence processes in *Arabidopsis* (Ren et al., 2017). As a result, the SA-mediated defense pathway is promoted, hence the resistance to bacteria and biotrophic fungi might increase, and the JA-mediated pathway is antagonized, increasing the susceptibility to necrotrophic pathogens. Solid arrows denote direct positive regulation of genes or pathways. “T” lines mean negative regulation. Dashed lines evidence positive correlation between plant events.

Based on the results described above for *AtWRKY75* and its homologs in plants, a tentative emerging proposal for the regulatory mechanism played by *AtWRKY75*-like genes in plant defense response in green tissues is described in Figure 6. It is worthwhile to note that this proposal only summarizes previous results described so far for AtWRKY75 and WRKY75-like genes in other plant species. According to this tentative model and being FaWRKY1 a WRKY75-like transcription factor, it is not unreasonable to think that FaWRKY1 also might be involved in the up-regulation of certain *JAZ* genes such as *FaJAZ5* and *FaJAZ9 in* strawberry. Therefore, when FaWRKY1 is present, a subsequent limited repression of genes involved in the early response of the JA-mediated pathway might be produced. Notably, under *C. acutatum* fruit infection the expression of *FaWRKY1* is upregulated [Figure 2, 5, and Encinas-Villarejo et al. (2009)] and upon this pathogen interaction, a partial activation of JA-defensive pathway has been previously described in strawberry (Amil-Ruiz et al., 2016). Also in strawberry, a correlation between JA-Ile levels and expression pattern of some JAZ encoding genes (*FaJAZ1/8.1*) during fruit development, and JA-treated fruit has been reported (Garrido-Bigotes et al., 2018a,b) opening the possibility that increase in JA-Ile content by pathogen attack could up-regulate JAZ expression irrespectively of the WRKY75-like mediated control. In addition, also similarly to AtWRKY75, FaWRKY1 might stimulate the biosynthesis of SA through upregulation of the *FaICS1* ortholog, which in turn will promote the activation of the SA-mediated pathway and the production of ROS. Notably, increases in SA and JA content has been described in strawberry after *C. acutatum* infection (Amil-Ruiz et al., 2016), and the ectopic expression of *FaWRKY1* in *Arabidopsis* wild-type and *WRKY75At22* mutant genetic backgrounds promoted the production of high levels of H_2_O_2_ after being challenged with *P. syringae* (Encinas-Villarejo et al., 2009).

In an attempt to address whether FaWRKY1 suits to this tentative model in strawberry fruit and expand our understanding of potential molecular players within this complex regulatory network, we have monitored the molecular signature of ICS1, CAT2, JAZ and WHY family orthologs in strawberry and in both *FaWRKY1* silenced and overexpressed fruit. Thus, we have identified one *FaICS1*, one *FaCAT* and two *FaWHY* genes in strawberry and twelve ortholog members of the JAZ protein family have previously been described and characterized in the diploid woodland strawberry *F. vesca* (Garrido-Bigotes et al., 2018a) (Supplementary Table S1). Only *FaCAT*, *FaWHY1*, *FaWHY2*, *FaJAZ9* and *FaJAZ5* genes responded positively to *C. acutatum* infection in non-agroinfiltrated fruit (Figure 5). Moreover, we could not detect significant changes in gene expression for any of the strawberry tested genes in fruit in which *FaWRKY1* was transiently silenced or overexpressed. Therefore, in our experimental conditions, no clear positive or negative correlation can be inferred between the FaWRKY1 and the strawberry *FaICS1, FaCAT, FaWHY*, or *FaJAZ* genes here analyzed and this matter remains to be further elucidated.

It is worth reminding that the non-climacteric strawberry fruit growing and ripening process is controlled by programmed hormonal changes, and it is different from other fruit species and plant tissues. Remarkably, an ABA increase in the red stage takes place (Symons et al., 2012). It has been shown that ABA mediates defense responses positive or negatively depending on the pathogen life style and tissue infected. Studies in *Arabidopsis* have shown that, after pathogen penetration, ABA antagonizes the SA-dependent defenses effective against biotrophic and hemibiotrophic pathogens. On the other hand, ABA promotes the MYC branch of the JA pathway while suppresses the ERF1/ORA59 branch, compromising the resistance to necrotrophs (Ton et al., 2009; Pieterse et al., 2012). In addition, changes in pH and carbon availability during ripening can modulate the expression of host genes and are main factors contributing to susceptibility of fruit to pathogen colonization, as reported in the tomato fruit-*C. gloeosporioides* interaction (Barad et al., 2017; Prusky and Wilson, 2018). Consequently, the expression pattern of defense related genes against *C. acutatum* and/or other pathogen infections could be modulated differently in this fruit tissue in comparison to other strawberry tissues.

In summary, in this paper a functional characterization of the *FaWRKY1* gene has been accomplished in strawberry fruit. We provide evidences that suggest the relevance between FaWRKY1 and strawberry fruit disease resistance against *C. acutatum*. Thus, FaWRKY1 act as a negative regulator of strawberry fruit resistance to *C. acutatum.* The FaWRKY1 responsive elements and molecular mechanisms involved in the defense response to *C. acutatum* remain elusive and further studies are still needed to unravel the intricate and complex regulatory network of FaWRKY1 in strawberry.

## Author Contributions

JH and JC conceived and designed the experiments. JC contributed to reagents, materials and analysis tools. JM, FP-A, and JM-B made experimental suggestions. JH, JG-G, IA-G, AL, CL-H, and JC carried out experiments. JH, JG-G, FA-R, IA-G, AL, CL-H, and JC analyzed and interpreted the data. JH, JG-G, and JC contributed to drafting the manuscript. JH, JG-G, FA-R, CL-H, JM, FP-A, JM-B, and JC provided a critical review. All authors approved the final version of the manuscript.

## Conflict of Interest Statement

The authors declare that the research was conducted in the absence of any commercial or financial relationships that could be construed as a potential conflict of interest.

## Footnotes

1 http://faostat.fao.org

2 http://www.real-statistics.com/
