# Supplementary material for: The Strawberry FaWRKY1 Transcription Factor Negatively Regulates Resistance to Colletotrichum acutatum in Fruit Upon Infection
Source: Front Plant Sci. 2019 Apr 18;10:480. doi: 10.3389/fpls.2019.00480 (PMC6482226; doi:10.3389/fpls.2019.00480)
Supplement: Supplementary file 1 [file Data_Sheet_1.PDF]

Supplementary Material

The strawberry FaWRKY1 transcription factor negatively regulates resistance to *Colletotrichum acutatum* in fruit upon infection

***A*uthors:** José Javier Higuera[1†], José Garrido-Gala[1†], Ayman Lekhbou[1], Isabel Arjona-Girona[2], Francisco Amil-Ruiz[3], José A. Mercado[4], Fernando Pliego-Alfaro[4], Juan Muñoz-Blanco[1], Carlos J. López-Herrera[2], José L. Caballero[1]*

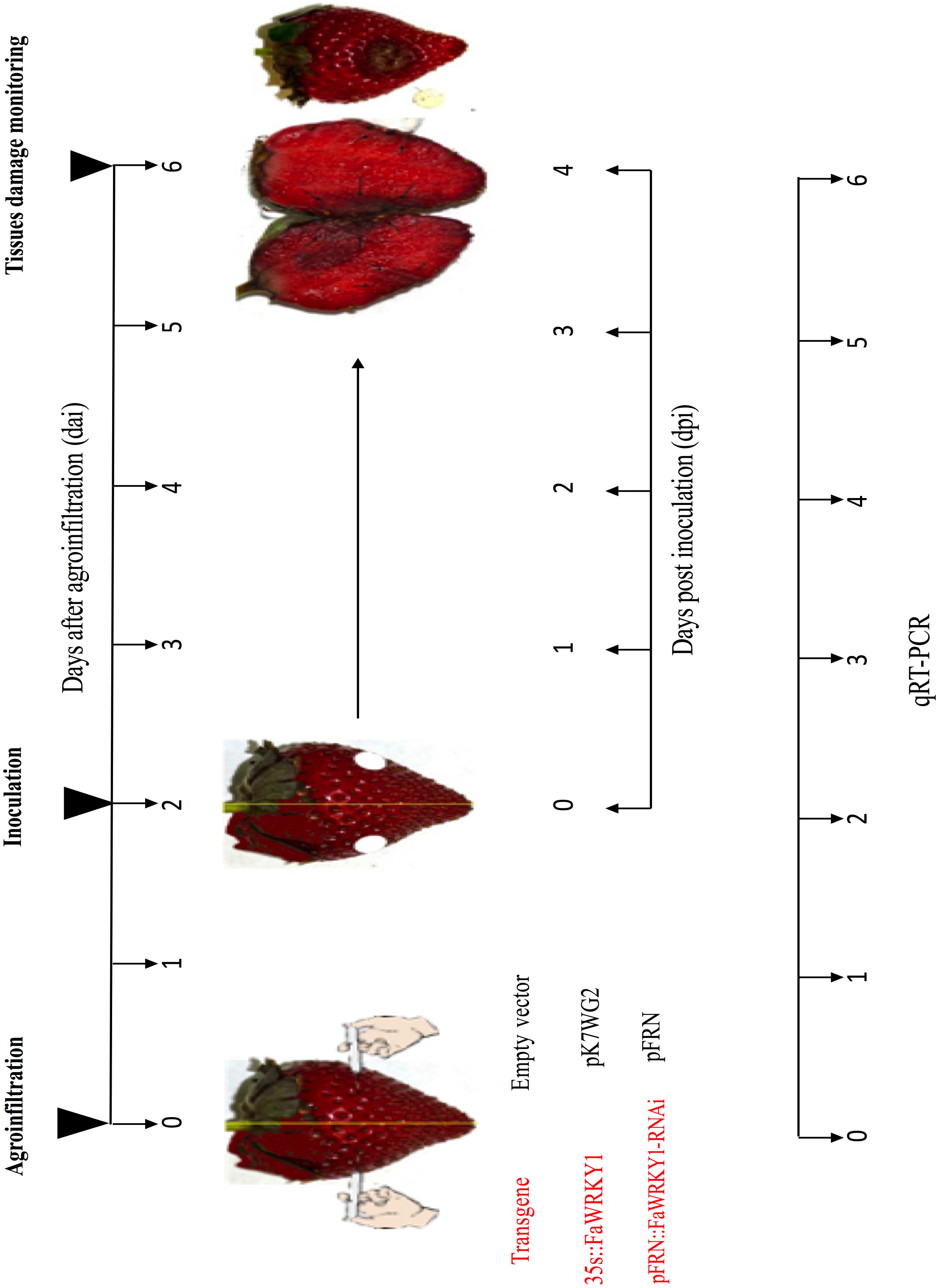

Supplementary Figure 1. Experimental procedure for tissue damage assessment produced by *C. acutatum* in strawberry fruits agroinfiltrated with AGL0 bearing overexpression or silencing FaWRKY1 gene constructs

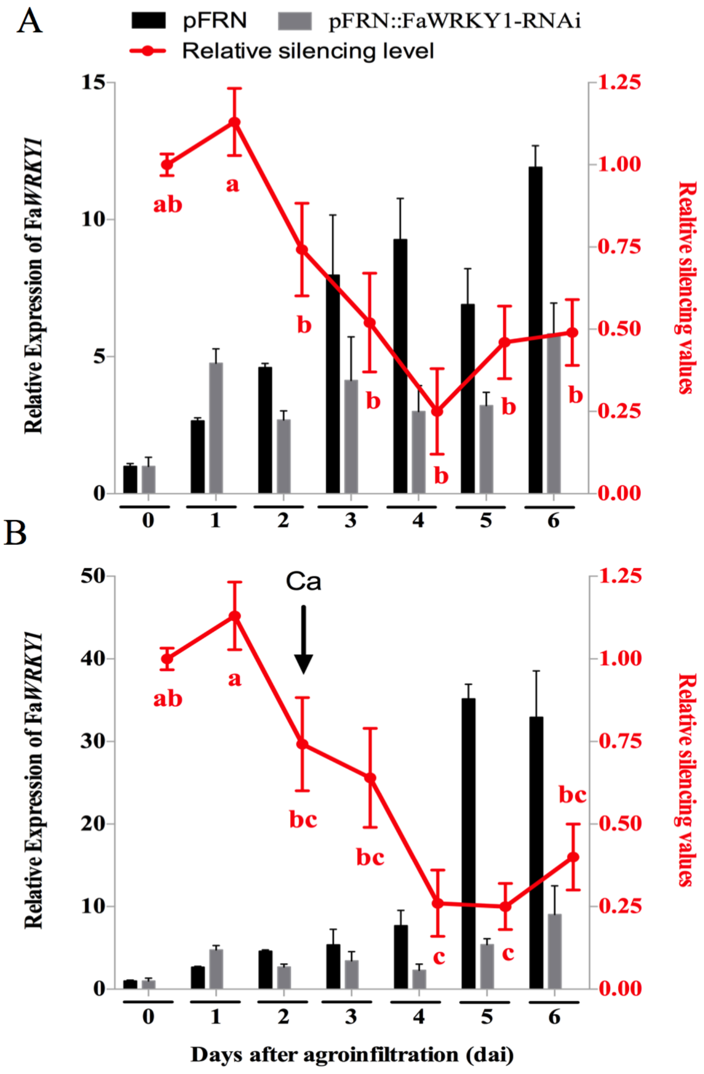

Supplementary Figure 2. Effect of transient silencing of FaWRKY1 gene in Strawberry Fruit by agroinfiltration and *C. acutatum* infection. Gene expression kinetic analysis of *FaWRKY1* by qRT-PCR in each of the two strawberry fruit halves agroinfiltrated with pFRN (black line) and pFRN::FaWRKY1-RNAi (grey line), respectively, and non-inoculated (A) or inoculated with *C. acutatum* (B). In the graphics, standard value 1 at T0 was added to better illustrate changes. Data were normalized with respect to the transcript level of the housekeeping *elongation factor 1α* and *actine 1* genes, and scored during six days. Values are the means of three biological replicates. In red, the level of FaWRKY1 silencing was calculated as a ratio value between the gene expression values found within the pFRN::FaWRKY1 fruit half with respect to the corresponding pFRN half. Means followed by the same letters in each trait are not significantly different at 5% level, according to tukey's test.

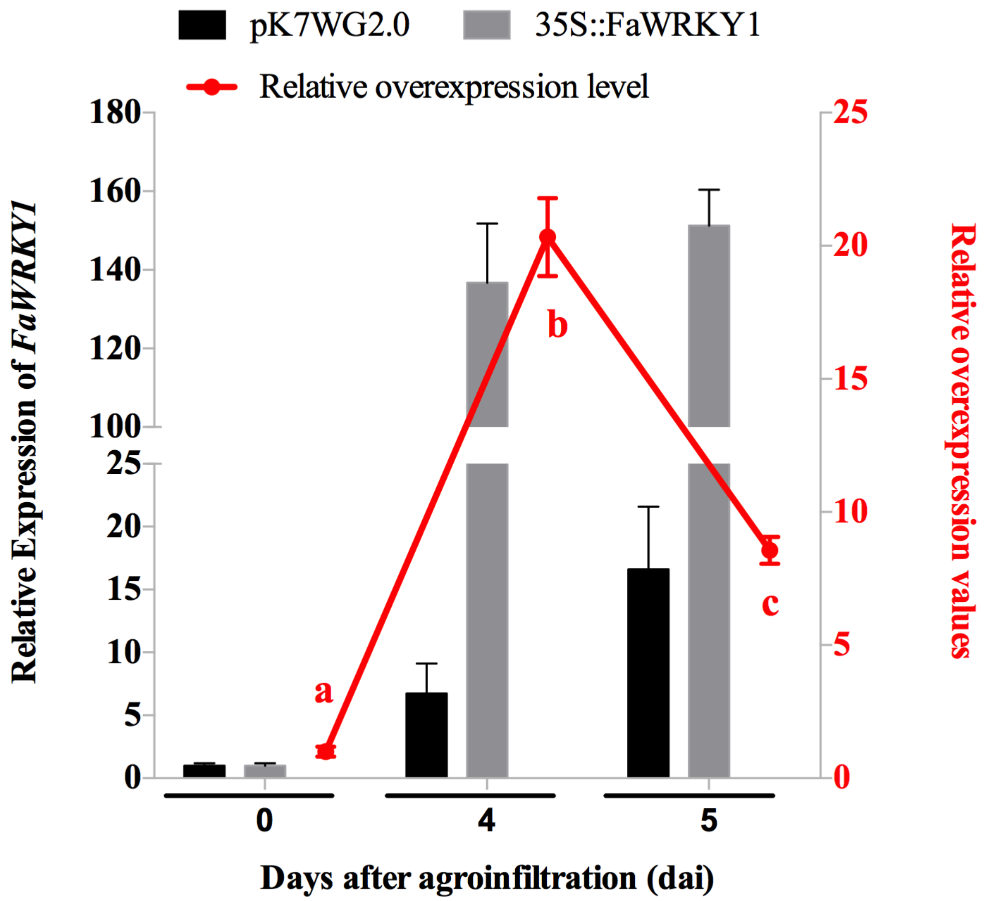

Supplementary Figure 3. Effect of transient ectopic expression of *FaWRKY1* gene in strawberry fruit by agroinfiltration and *C. acutatum* infection. Gene expression kinetic analysis of *FaWRKY1* by qRT-PCR in each of the two strawberry fruit halves agroinfiltrated with pK7::WRKY1 (35s::FaWRKY1) (grey line) or pK7GW2.0 (black line), respectively, and inoculated with *C. acutatum*. In the graphics, standard value 1 at T0 was added to better illustrate changes. Data were normalized with respect to the transcript level of the housekeeping elongation factor 1α and actine 1 genes. Values are the means of three biological replicates. In red, the level of overexpression of *FaWRKY1* was calculated over time as a ratio value between the gene expression values found within the pK7::WRKY1 fruit half with respect to the corresponding pK7WG2.0 half. Means followed by the same letters in each trait are not significantly different at 5% level, according to tukey's test.

Table S1. Primers and sequences used in this study.

Name	Description / Orthologs		Sequence (5´ - 3´)	Observations
FaWRKY1*	WRKY DNA-binding protein 75	sense chain	ACAGCAGTAAGATTAGGGATGAAGAAGGGAG	Amil-Ruiz et al., 2013 **<br>Amil-Ruiz et al., 2016 *<br>RT-qPCR
	Fv gene07210 / AT5G13080	anti-sense chain	GCTTCTTCACATTGCAACCCTGATGCGTG	
FaCHI4-2*	Chitinase class IV	sense chain	TGCCGGCAAGAGCTTCTACACTAGACAG	
	Fv gene02717 / AT3G54420	anti-sense chain	TGTGAAGGATGTGTCGCAGTAGGTGG	
FaACTIN**	Actin	sense chain	GGGCCAGAAAGATGCTTATGTCGG	
	Fv gene26612 / AT3G12110	anti-sense chain	GGGCAACACGAAGCTCATTGTAGAAG	
FaEF1a**	Elongation factor 1-alpha	sense chain	TGGATTTGAGGGTGACAACATGA	
	Fv gene28639 / AT5G60390	anti-sense chain	GTATACATCCTGAAGTGGTAGACGGAGG	
FaICS1	ICS1	sense chain	TTGGTCAGTGTTGCTGGTGT	This work<br>RT-qPCR
	Fv gene25950 / AT1G74710	anti-sense chain	GTAGCCAAAGGCCTCCCATT	
FaCAT	Catalase 2	sense chain	CCTGCCCTTATTGTCCCTGG	
	Fv gene10917 / AT4G35090	anti-sense chain	GTGAGCAGACTTGGGAGCAT	
FaWHY1	Whirly1	sense chain	TTTCGGAAGAGACGACTCGC	
	Fv gene04012 / AT1G14410	anti-sense chain	ACATAAAACCTAGCCGGCCC	
FaWHY2	Whirly2	sense chain	ACACAAAGCACCGGCTTTTC	
	Fv gene31174 / AT1G71260	anti-sense chain	AGAGAGTGCAGCTTTGCCTT	
FaJAZ1	*FvTIFY10A*	sense chain	TGGGAGATCTGAACCTCGTC	Garrido-Bigotes et al., 2018<br>RT-qPCR
	Fv gene12541 / AT1G19180	anti-sense chain	TTCCTCGGTTTCTCCATCAC	
FaJAZ4	*FvTIFY6B*	sense chain	AGAAGTGCTGGTGCACATTG	
	Fv gene06180 / AT3G17860	anti-sense chain	TGGGCATAAATCTGGAGGAC	
FaJAZ5	*FvTIFY11A-Like*	sense chain	CACCATGAACTTGCTCAACG	
	Fv gene05383 / AT1G17380	anti-sense chain	GAAAGGTCGCTGAAGACGAG	
FaJAZ7	*FvTIFY5B*	sense chain	GGATGAGCAGACCAGACAGG	
	Fv gene24321 / AT2G34600	anti-sense chain	AAACATAAACCCGGCCATCG	
FaJAZ8.1	*FvTIFY5A*	sense chain	GAGGAGGAACTGCAATTTGG	
	Fv gene30624 / AT1G30135	anti-sense chain	AAGAGGGAAGCCGGAATTAG	
FaJAZ9	*FvTIFY6B*	sense chain	GGATGAGCAGACCAGACAGG	
	Fv gene09356 / AT5G13220	anti-sense chain	AAACATAAACCCGGCCATCG	
FaJAZ10	*FvTIFY9*	sense chain	TTCCAGAAGTTCCTCGAACG	
	Fv gene07265	anti-sense chain	GATTTCCTGGCTGCAATCAC	
FaJAZ12	*FvTIFY3B*	sense chain	GAAGCGTAGGGACAGATTGG	
	Fv gene12975 / AT5G20900	anti-sense chain	AACCGGAAGAAGCATCATTG	
**pFRN::FaWRKY1 (RNAi)**	FaWRKY1-RNAi	sense chain	ATGGATACCTACCCAGCATTCTA	Cloning of a 272 bp fragment from *FaWRKY1* in pFRN
		anti-sense chain	TCCCTTCTTCATCCCTAATC	
**35S::FaWRKY1 (Overexpression)**	WRKY DNA-binding protein 75	sense chain	GGGGACAAGTTTGTACAAAAAAGCAGGCTTCAACAA TGGATACCTACCCAGCATTCTA	Encinas-Villarejo et al., 2009
		anti-sense chain	GGGGACCACTTTGTACAAGAAAGCTGGGTCTTACAA AGCACGCACAGCAGGCA	
**pCaMV35s::GUS**	Cauliflower mosaic virus (CaMV) promoter	sense chain	GGGGACAAGTTTGTACAAAAAAGCAGGCTACTAGAG CCAAGCTGATCTCCTT	Cloning of CaMV promoter (1035 bp) in pKGWFS7.0
		anti-sense chain	GGGGACCACTTTGTACAAGAAAGCTGGGTTCGACTA GAATAGTAAATTGTAATG	
**FaWRKY1-RNAi**	WRKY DNA-binding protein 75		ATGGATACCTACCCAGCATTCTATTCTTCTTCATCAACACCACCTTCTGCT GCTGCTTCTTCGCTGTCATTGAACATGGTGAACACTCATCCTCATCATGCT TACGGTAACGATCATCAGTACCAAGCTAGCAATAACAAGGAAAATGGGTT CTTGGGGCTGATGTCAGAGATGGAGGTTTCGAACAGCATGAGTAGTATTAC CCAGCAGAGTATGAAAAGCTTTGGGGAGGGTGAAAGTAATACAGCAGTAA GATTAGGGATGAAGAAGGGA	*FaWRKY1* fragment of 272 bp for RNAi gene silencing

Table S2: Distribution of fruit number according to season and constructs used in the present study

		1º year		2º year		Total	
**Construct**	**Purposes**	**+I**	**-I**	**+I**	**-I**	**+I**	**-I**
pFRN::FaWRKY1-RNAi	Expression	24	24	-	-	120	24
	Statistic	48	-	48	-		
pFRN (RNAi control)	Expression	24	24	-	-	120	24
	Statistic	48	-	48	-		
	Total per year	192		96		288	
35S::FaWRKY1	Expression	24	24	-	-	120	24
	Statistic	48	-	48	-		
pK7WG2 (OE control)	Expression	24	24	-	-	120	24
	Statistic	48	-	48	-		
	Total per year	192		96		288	

Fruits were inoculated with *C.acutatum* **(+I)** or non-inoculated **(-I).**
